# The PROTEA trial: darunavir/ritonavir with or without nucleoside analogues, for patients with HIV-1 RNA below 50 copies/mL

**DOI:** 10.7448/IAS.17.4.19525

**Published:** 2014-11-02

**Authors:** Andrea Antinori, Jose Arribas, Jan Fehr, Pierre-Marie Girard, Andrzej Horban, Andrew Hill, Yvon van Delft, Christiane Moecklinghoff, Andrew Hill

**Affiliations:** 1 Infectious Diseases, National Institute of Infectious Diseases, Rome, Italy; 2 IdiPAZ, Hospital la Paz, Madrid, Spain; 3 Infectious Diseases, University Hospital Zurich, Zurich, Switzerland; 4 Maladies Infectiueses et Tropicales, Hôpital Saint-Antoine, Paris, France; 5 Infectious Diseases, Warsaw Medical University, Warsaw, Poland; 6 Janssen, R&D, High Wycombe, UK; 7 Janssen, EMEA, Tilburg, Netherlands; 8 Janssen, EMEA, Neuss, Germany; 9 Pharmacology and Therapeutics, University of Liverpool, Liverpool, UK

## Abstract

**Introduction:**

In previous studies, protease inhibitor (PI) monotherapy has shown trends for higher low-level elevations in HIV-1 RNA compared to triple therapy, but no increase in the risk of drug resistance.

**Methods:**

A total of 273 patients with HIV-1 RNA <50 copies/mL for over 24 weeks on current antiretrovirals switched to DRV/r (darunavir/ritonavir) 800/100 mg once-daily, either as monotherapy (*n*=137) or with 2NRTIs (nucleoside reverse-transcriptase inhibitors) (*n*=136), after a 4 week run-in phase with DRV/r + 2NRTI. Treatment failure was defined as HIV-1 RNA levels above 50 copies/mL (FDA Snapshot method) by Week 48, or switches off study treatment. Patients with elevations in HIV-1 RNA on DRV/r monotherapy could be re-intensified with NRTIs. The trial had 80% power to show non-inferiority for the monotherapy arm (delta = − 12%).

**Results:**

Patients were 83% male and 87% Caucasian, with mean age 42 years; 10% were HCV antibody positive. In the DRV/r monotherapy arm, there were more patients with nadir CD4 count below 200 cells/µL (30% versus 22%). In the primary efficacy analysis, HIV-1 RNA <50 copies/mL by Week 48 (intent-to-treat (ITT)) was 118/137 (86.1%) in the DRV/r monotherapy arm versus 129/136 (94.9%) in the triple therapy arm; DRV/r monotherapy did not show non-inferiority versus triple therapy in the primary analysis (difference=− 8.7%, 95% CI −15.5 to −1.8%). In the multivariate analysis, the main predictor of treatment failure was nadir CD4 count. For patients with nadir CD4 counts <200 cells/µL, HIV-1 RNA suppression rates at Week 48 were 27/41 (66%) in the DRV/r monotherapy arm and 29/30 (97%) in the triple therapy arm; for patients with CD4 nadir at least 200 cells/µL, HIV-1 RNA suppression rates were 91/96 (95%) in the DRV/r monotherapy arm and 100/106 (94%) in the triple therapy arm. In the overall population, by a switch included analysis, efficacy was 92.0% versus 96.3%, showing non-inferiority (difference=− 4.3%, 95% CI=−9.7 to +1.2%). No treatment-emergent primary PI mutations were detected in three patients with sustained elevations in HIV-1 RNA at least 400 copies/mL (two on PI monotherapy, one on triple therapy). CD4 counts remained stable during the trial in both arms.

**Conclusions:**

In this study for patients with HIV-1 RNA < 50 copies/mL at baseline, switching to DRV/r monotherapy showed lower efficacy versus triple antiretroviral therapy at Week 48 in the primary switch equals failure analysis (86% versus 95%). However, this lower efficacy was seen mainly in patients with CD4 nadir levels below 200 cells/µL. There was no development of PI resistance.

